# Deep learning tomographic reconstruction through hierarchical decomposition of domain transforms

**DOI:** 10.1186/s42492-022-00127-y

**Published:** 2022-12-09

**Authors:** Lin Fu, Bruno De Man

**Affiliations:** GE Research, NY 12309 Niskayuna, USA

**Keywords:** Computed tomography, Image reconstruction, Deep learning, Hierarchical

## Abstract

Deep learning (DL) has shown unprecedented performance for many image analysis and image enhancement tasks. Yet, solving large-scale inverse problems like tomographic reconstruction remains challenging for DL. These problems involve non-local and space-variant integral transforms between the input and output domains, for which no efficient neural network models are readily available. A prior attempt to solve tomographic reconstruction problems with supervised learning relied on a brute-force fully connected network and only allowed reconstruction with a 128^4^ system matrix size. This cannot practically scale to realistic data sizes such as 512^4^ and 512^6^ for three-dimensional datasets. Here we present a novel framework to solve such problems with DL by casting the original problem as a continuum of intermediate representations between the input and output domains. The original problem is broken down into a sequence of simpler transformations that can be well mapped onto an efficient hierarchical network architecture, with exponentially fewer parameters than a fully connected network would need. We applied the approach to computed tomography (CT) image reconstruction for a 512^4^ system matrix size. This work introduces a new kind of data-driven DL solver for full-size CT reconstruction without relying on the structure of direct (analytical) or iterative (numerical) inversion techniques. This work presents a *feasibility* demonstration of full-scale learnt reconstruction, whereas more developments will be needed to demonstrate *superiority* relative to traditional reconstruction approaches. The proposed approach is also extendable to other imaging problems such as emission and magnetic resonance reconstruction. More broadly, hierarchical DL opens the door to a new class of solvers for general inverse problems, which could potentially lead to improved signal-to-noise ratio, spatial resolution and computational efficiency in various areas.

## Introduction

**Fig. 1:**
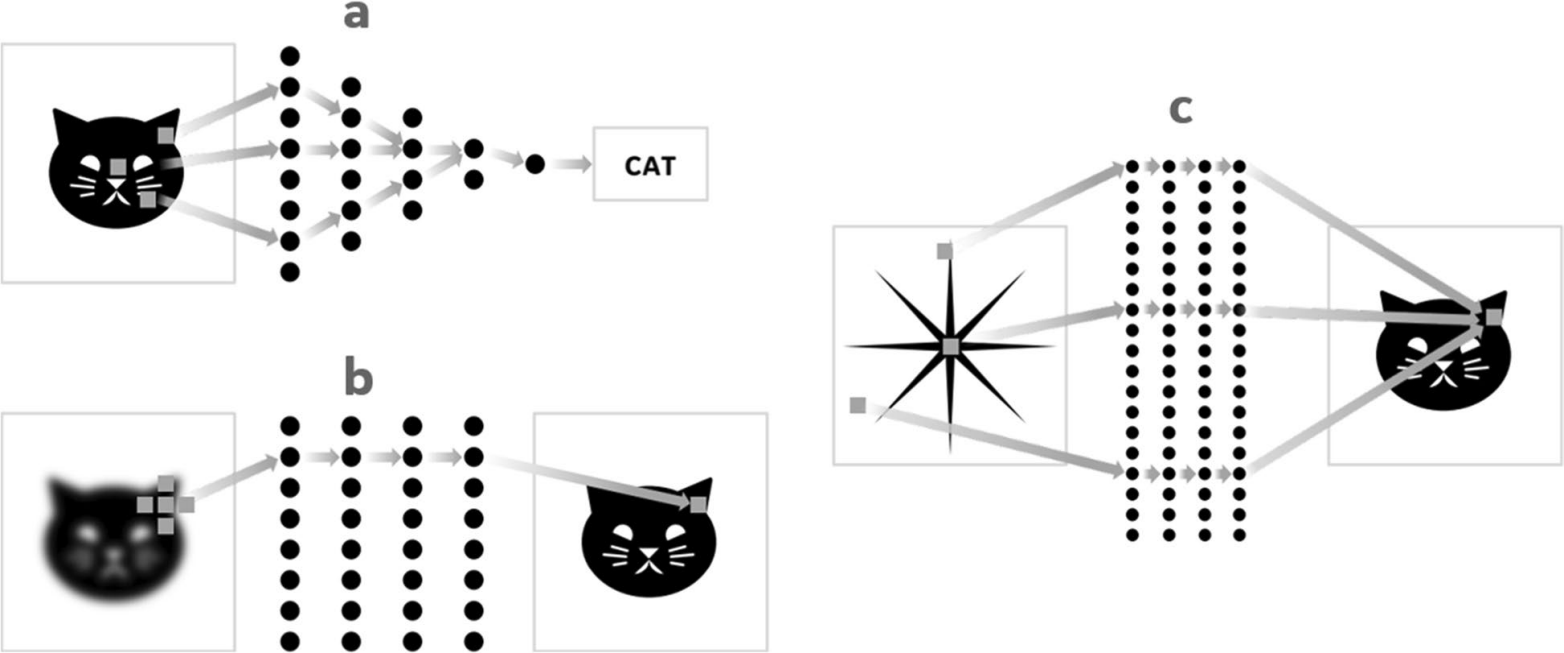
Schematic representation of DL for three categories of problems: image analysis (**a**), image enhancement (**b**) and domain transforms (such as Fourier transform) (**c**). Domain transform remains challenging for DL because there is no direct spatial linkage between the input and output domains, hence large-scale problems are usually infeasible to a neural network
- *Image enhancement* applications aim to improve the image in some context-dependent way. The input is an image; the output is another image (Fig. 1b). Input and output images are spatially ‘linked’ so the network exhibits a high degree of locality – input values mostly affect only output values in their immediate vicinity – hence training is commonly performed with small image patches. In this category, typical applications include image sharpening [8], image denoising [9–11], and semantic segmentation [12, 13].

The surge in deep learning (DL) imaging research in recent years has resulted in a plethora of applications and network architectures [1–5]. Most of these approaches can be categorized in two major areas:

- *Image analysis* applications seek to make a decision or diagnosis. The input to the DL network is an image and the output is a discrete set of labels (Fig. 1a). All the voxels in the input image are indirectly linked to the final labels through a complex neural relationship. Examples of this category include the classification of images as cats and dogs [6] and the diagnosis of lesions in medical images as malignant or benign [7].

The focus of this paper will be a third category of applications – *domain transforms*. In these problems both input and output are relatively large datasets represented as two- or higher-dimensional arrays, but without a local spatial linkage between the two (Fig. 1c). This is the case for many inverse problems. The transform between the two domains may take the form of an integral transform and the spatial relationship between the input and output may be non-local and shift-variant. Each input datum contributes to many output data and – vice versa – each output datum is defined by many input data. This category includes the Fourier transform [14], tomographic reconstruction [15], and many inverse problems in geoscience, physics, healthcare, and defense applications.

The challenge with this third category is the very high dimensionality of the neural networks. Unlike the other two categories of applications, it can be difficult or infeasible to train a network for the third category of applications. In matrix terminology, the absence of simple spatial linkage results in non-sparse matrices or non-convolutional matrices, making conventional neural network models less effective. A brute-force network model of such problems would result in an extremely high-dimensional network, which poorly generalizes to large-scale problems. For example, using a dense network to model the transformation between two-dimensional (2D) images of size 512 × 512 would result in a number of network weights on the order of 512^4^ ≈ 64 billion. This also explains why prior attempts to address the third category of problems using DL are typically limited to 64 × 64 or 128 × 128 images [14, 15].

Here we present a hierarchical DL approach for the third category of problems along with a more broadly applicable hierarchical DL framework. Instead of using a brute-force neural network to model a high-dimensional and non-local domain transform, the proposed approach decomposes the transform into hierarchical stages via a sequence of intermediate data representations, where the transforms between the intermediate representation only employ local operations, which can be efficiently mapped to neural networks. Such hierarchical decomposition leads to exponentially fewer parameters than a fully connected network would need. In matrix terminology, this is analogous to approximating a dense matrix by a product of sparse matrices. In addition, we introduce the idea of training DL networks with computer simulated random noise patterns, which overcomes the need for large amount of training data for supervised learning.

We apply the hierarchical DL framework to computed tomography (CT) reconstruction to show its feasibility in full-scale inverse problems. Until recently, all CT reconstruction algorithms could be categorized as direct reconstruction or iterative reconstruction. Direct reconstruction approaches such as filtered backprojection (FBP) [16–18] have relatively low complexity and result in perfect images under strongly idealized conditions. Iterative reconstruction approaches [19–21] can more effectively deal with noise and other non-idealities but have high computational complexity. The hierarchical DL framework proposed here replaces the entire domain transform by a data-driven learned inversion, and it is a radical change that does not rely on a conventional direct inversion or on an iterative data fit optimization. In other words, direct reconstruction relies on a mathematical inversion of a Radon transform or a cone-beam transform, and iterative reconstruction relies on numerical inversion of the same, while the proposed hierarchical reconstruction is a supervised learning method that belongs to neither of them. The use data-driven inversion techniques for image reconstruction opens the door to an entirely new third type of reconstruction [2].

To our knowledge, only a few groups have attempted to realize such pure data-driven DL CT reconstruction [15, 22]. The first and most prominent example is AUTOMAP [15], which is based on dense networks and thus are difficult to scale to full-size data. The hierarchical approach we present here was conceived independently of the AUTOMAP approach [23, 24]. Based on a novel hierarchical decomposition of the domain transform, the proposed approach has the advantage of being fundamentally scalable to full-size problems. In comparison, most other existing approaches that use DL for image reconstruction are limited to augmenting conventional reconstruction algorithms instead of replacing them, due to the high dimensionality associated with modeling domain transforms by neural networks. They often rely on a conventional reconstruction algorithm to produce an initial image or a conventional forward/back projection operator to perform the domain transform. Such DL-augmented signal processing components have been used in acceleration of iterative reconstruction [25], post-reconstruction image denoising or restoration [9–11, 26], improved prior functions for Bayesian reconstruction [27, 28], unrolled iterative reconstruction [5, 29, 30], optimization of projection or image-domain filter weights [3], DL-based reconstruction with FBP-like processing pipeline [31–33] or stacked analytical backprojection [34], and raw data analysis to bypass reconstruction entirely [35]. However, these efforts did not aim to replace the entire domain transform by a data-driven supervised learning approach, and they still rely – at least in part – on the pipeline of conventional algorithms.

Since the proposed hierarchical reconstruction is a novel framework for modeling domain transforms, this paper focuses on the theoretical development and is limited to a feasibility study to demonstrate scalability to full-scale 2D CT problems, as well as image quality equivalent with FBP reconstruction. In future work, the proposed computationally efficient networks can be augmented to model rich data-driven prior information and compete with state-of-the-art iterative methods or DL-augmented reconstructions in terms of image quality and dose-efficiency. The hierarchical framework is extendable to three dimensions and to other applications such as magnetic resonance reconstruction and emission reconstruction, although these extensions are not implemented within the scope of this paper.

It is worth noting that the proposed hierarchical reconstruction framework is not related to the previously published hierarchical projection and backprojection approach [36]. The latter is a fast, numerical implementation of a traditional component and is used as part of a direct or iterative reconstruction algorithm, while the proposed technique is an entirely new reconstruction framework.

## Methods

### General theory

Consider inverse problems whose forward models can be written in the form of the Fredholm integral equation of the first kind

1 $$p\left(v\right)= \int K\left(u,v\right)f\left(u\right)\mathrm{d}u$$

The goal is to infer the function $f(u)$, given the kernel function $K(u,v)$ and the observation $p(v)$. When the kernel $K(u,v)$ is non-local and space-variant, it is often difficult or impractical to model the solution with a neural network, especially for large-scale problems.

A complicated transform can sometimes be decomposed into a sequence of simpler hierarchical steps, providing an avenue for fast and efficient algorithms. Here we propose a new framework to make such problems more amenable to data-driven supervised learning. Since the major challenge of the problem are the non-local kernels (hence a non-sparse network), we introduce a virtual intermediate data domain by applying a window function $\Pi (\cdot )$ to the integration

2 $${q}_{\alpha }\left(v,t\right)=\int K\left(u,v\right)f\left(u\right)\Pi (\frac{t-u}{\alpha })\mathrm{d}u$$

where $\Pi \left(x\right)$ is a window function and $\Pi \left(x\right)=1$ for $\left|x\right|<0.5$ and $\Pi \left(x\right)=0$ elsewhere, $\alpha$ is a scale factor controlling the window size, and $t$ is a parameter specifying the location of the window. By varying $\alpha$, a continuum of intermediate representations between $p\left(v\right)$ and $f(u)$ (like a homotopy) can be defined. Both $p(v)$ and $f(u)$ can be viewed as marginal cases of ${q}_{\alpha }(v,t)$. In the limiting case that $\alpha \to 0$, the window becomes a Dirac delta impulse, thus $\underset{\alpha \to 0}{\mathrm{lim}}{q}_{\alpha }\left(v,t\right)=K(t,v)f\left(t\right)$ and the integral vanishes. On the other hand, $\alpha \to \infty$ corresponds to an infinite window, making ${q}_{\alpha }\left(v,t\right)$ equal to the original measurement $p\left(v\right)$.

Although ${q}_{\alpha }\left(v,t\right)$ represents virtual data that cannot be physically measured, we propose that they can be synthesized and serve as intermediate steps in DL reconstruction. With progressively smaller integration window, ${\alpha }_{1}>{\alpha }_{2}>\dots >{\alpha }_{N}$, the original problem may be decomposed into a series of incremental steps

3 $$p\left(v\right)\to {q}_{{\alpha }_{1}}\left(v,t\right)\to {q}_{{\alpha }_{2}}\left(v,t\right)\to \dots \to {q}_{{\alpha }_{N}}\left(v,t\right)\to f\left(u\right)$$

Here ${q}_{\alpha }(v,t)$ represents a continuum between $p\left(v\right)$ and $f\left(u\right)$, and the incremental transitions from ${q}_{{\alpha }_{n}}\left(v,t\right)$ to ${q}_{{\alpha }_{n+1}}\left(v,t\right)$ possess a high degree of locality like a conventional image enhancement task. This gives rise to a hierarchical DL architecture that can tackle original domain transform problems by breaking them down to a hierarchy of simpler problems that are well-suited for implementation with neural networks.

### Hierarchical CT reconstruction

Here we present a detailed example of applying the hierarchical framework for solving the problem of domain transform in CT reconstruction. The purpose of CT reconstruction is to infer an image of the internal structure of an object from projection measurements taken along various rays passing through the object. The CT forward model can be expressed in the form of the Fredholm equation, with the kernel $K\left(u,v\right)=\delta \left({u}_{x}\mathrm{cos}{v}_{\theta }+{u}_{y}\mathrm{sin}{v}_{\theta }-{v}_{r}\right)$, where $\delta (\cdot )$ is the Dirac delta function, $u=({u}_{x},{u}_{y})$ are the image-domain coordinates, and $v=({v}_{r},{v}_{\theta }$) are the projection-domain coordinates.. The kernel represents projection rays parameterized by an offset $r$ and an angle $\theta$ in a 2D plane.

**Fig. 2:**
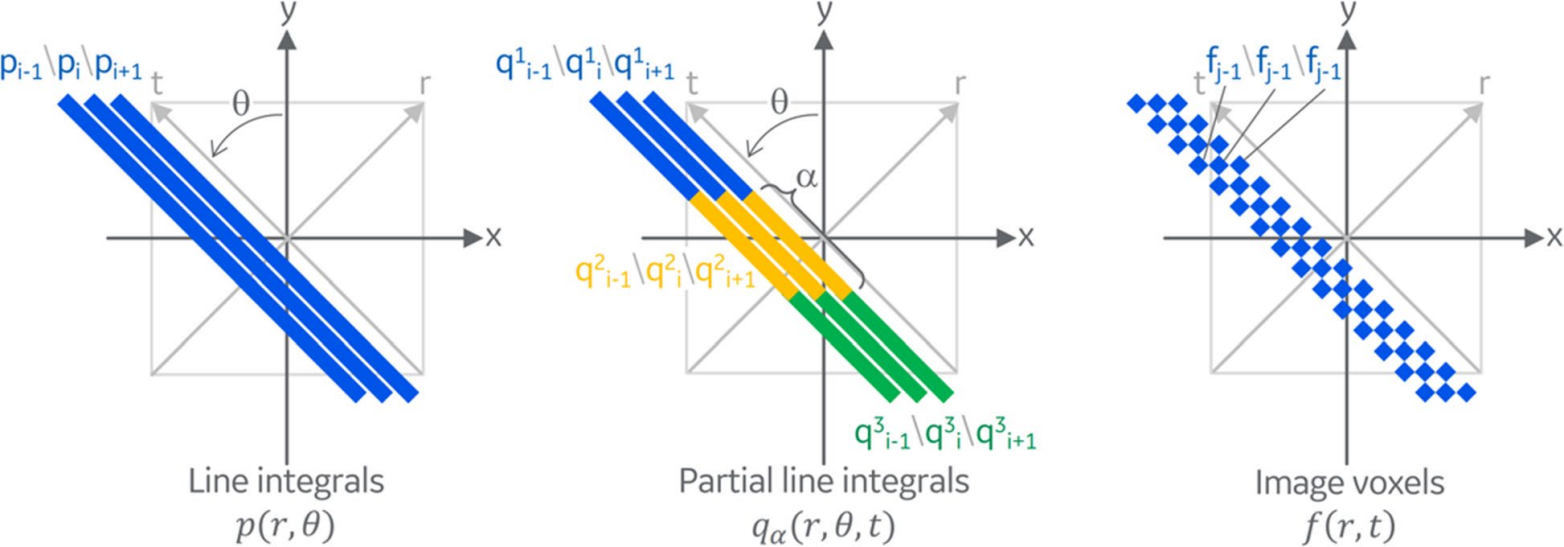
Partial line integrals are proposed as an intermediate representation between line integrals and image voxels, both of which being its marginal cases. The original CT measurements are line integrals over the entire length of the object. The reconstructed image is represented by the image voxels, which are essentially line integrals over the length of the voxel (except for a typical scale factor normalizing by the voxel size)

We define three data domains for hierarchical CT reconstruction (Fig. 2). First, the original CT measurements $p(r,\theta )$, namely the projections or sinogram, reside in the **line integral domain**. They represent the line integrals with the original kernel over the entire length of the object. We introduce a coordinate system *x–y* and a second coordinate system *r–t*, rotated by $\theta$. The projection lines are parametrized by a radial distance $r$ and a rotation angle $\theta$, and in the three-dimensional (3D) case, also by a *z*-distance (or cone angle). There is no depth resolution along *t* since the line integrals are over the entire projection lines. Second, the reconstructed image $f(x,y)$ is represented in the regular **voxel domain**. A voxel value can be interpreted as a marginal case of a line integral, where the integral is only over the length of the voxel. The voxel values are parametrized by $x$- and *y*-coordinates or by *r*- and *t*-coordinates, but there is no angular parameter because all voxels share the same angle; and in the 3D case also by a $z$-coordinate. Finally, the proposed intermediate data ${q}_{\alpha }(r,\theta ,t)$ are defined in the **partial line integral domain**, generated by restricting the line integral with a window function $\Pi (\frac{-{u}_{x}sin\theta +{v}_{y}\mathrm{cos}\theta -t}{\alpha })$ which corresponds to a line segment of length $\alpha$ centered at position $t$ along the projection line. The partial line integrals are parameterized by a radial distance $r$, an in-plane rotation angle $\theta$ (but typically fewer rotation angles than in the line integral domain), and a depth $t$. The partial line integral is also associated with the parameter $\alpha$, which specifies the width of the window function. Both the line integrals and the reconstructed voxels can be viewed as marginal cases of the partial line integrals.

**Fig. 3:**
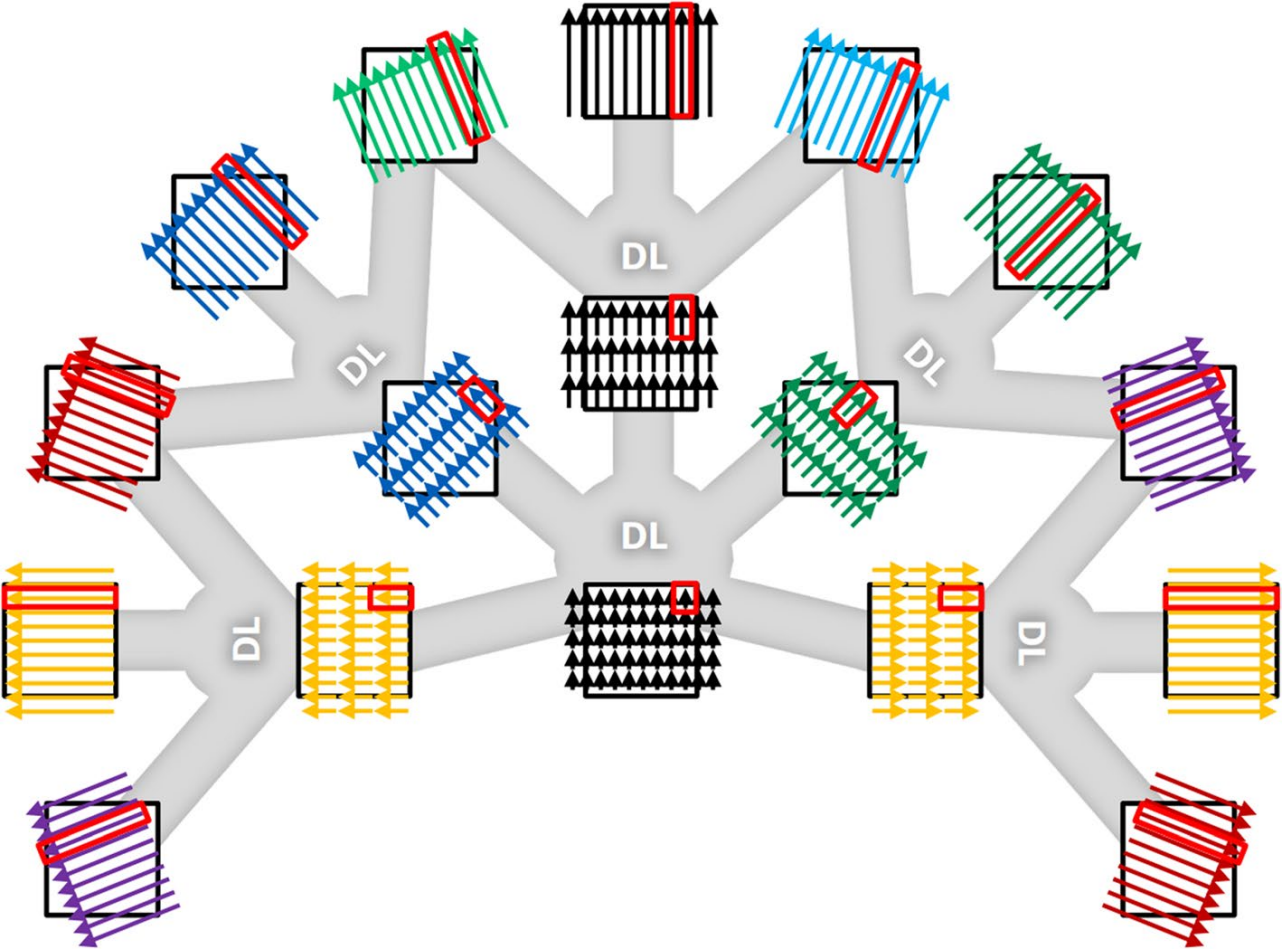
Illustration of the concept of hierarchical CT reconstruction. The diagrams in the outer ring represent the input data, i.e., line integrals at various rotation angles and radial offsets. The diagrams in the middle ring represent data in the intermediate domain, i.e., partial line integrals. The diagram at the center represents the output, i.e., the reconstructed image. The gray connections illustrate the flow of data through the network. As data go through the hierarchy, the depth resolution improves, while the number of angles decreases. The final reconstructed image is formed when the number of depth bins equals the desired size of the reconstructed image, and the number of angles reaches unity. The key benefit of hierarchical reconstruction is that the elementary reconstruction steps in each hierarchical stage are relatively localized, making it suitable for efficient implementation as neural networks. The red rectangles illustrate the localized correspondence across hierarchical stages

With the intermediate domain defined, CT reconstruction may be viewed as a process that progressively solves for partial line integrals with shorter and shorter integration length, until regular image voxels are reached.

4 $$p\left(r,\theta \right)\to {q}_{{\alpha }_{1}}\left(r,\theta ,t\right)\to {q}_{{\alpha }_{2}}\left(r,\theta ,t\right)\to \dots \to {q}_{{\alpha }_{N}}\left(r,\theta ,t\right)\to f\left(r,t\right){\left. \right|}_{\theta =0}$$

where ${\alpha }_{1}>{\alpha }_{2}>\dots >{\alpha }_{N}.$ In the last step, the rotation angle $\theta$ is sampled at angle zero so that the radial and depths coordinates $(r,t)$ are identical to the spatial coordinates $(x,y)$. In the marginal case that ${\alpha }_{N}$ reaches the desired size of the regular voxel, the partial line integral representation $f(r,t)$ becomes identical to a regular reconstructed image $f(x,y)$. Such a decomposition of tomographic reconstruction into incremental stages gives rise to a novel hierarchical flow for CT reconstruction (Fig. 3). The input data are line integrals with various offsets and orientations, but without depth resolution. Then, the proposed network transforms line integrals into partial line integrals, gaining depth resolution, while the number of angular sampling is reduced. Multiple intermediate stages with progressively finer depth resolution may be used (although only a single intermediate stage is shown in Fig. 3). Finally, the partial line integrals are transformed into regular image voxels as output, further gaining depth resolution. Overall, as data go through the hierarchy, the depth resolution increases, while the angular sampling density decreases, keeping the total amount of data approximately unchanged. Such a reconstruction may be viewed as a process that gradually trades angular resolution in the original sinogram for better depth resolution in a partial line integral domain and ultimately, generates the reconstructed image. Intuitively, the incremental elementary reconstruction step is analogous to a limited-angle tomosynthesis reconstruction, where a coarse level of depth resolution can be estimated from only a few projections in adjacent angles. In hierarchical reconstruction such incremental reconstruction step is repeated to incorporate information from wider and wider angular ranges and ultimately produces a regular reconstructed image with isotropic spatial resolution.

The key benefit of this hierarchical framework is that the elementary reconstruction from one stage to the next is localized in terms of number of angular ranges, number of radial distances and number of depth positions considered. The estimation of the partial line integrals only requires the line integrals that are at nearby angular positions and nearby radial positions. Similarly, the estimation of the voxel values requires as inputs only the partial line integrals that are at nearby radial and depth positions (indicated by red rectangles in Fig. 3). Hence the hierarchical framework decomposes a large-scale, non-local transform in a series of incremental local transforms, making the algorithm suitable for efficient implementation with a neural network. In matrix terminology, this decomposition is analogous to factorization of a large-scale dense matrix as the product of a series of sparse matrices.

**Fig. 4:**
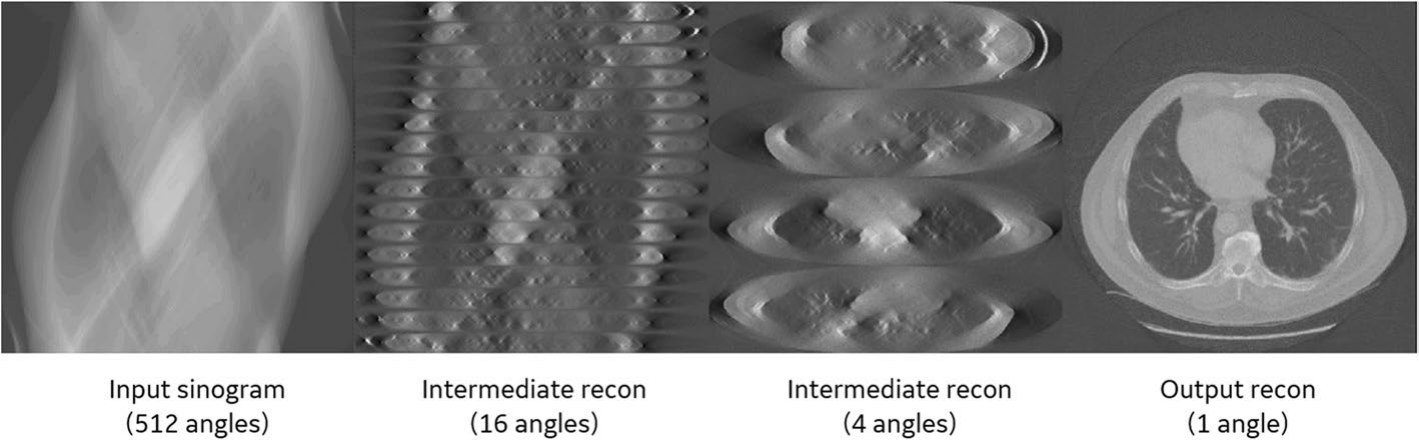
The progression of reconstruction through hierarchical stages. Two intermediate reconstructions are shown. All partial images at different rotation angles are stacked vertically for better visualization

Figure 4 shows the actual outputs from a hierarchical reconstruction network at different hierarchical stages for a realistic CT reconstruction example. (More details about the network architecture and training are provided in the next sections). The intermediate reconstructions are outputs from intermediate hierarchical layers and illustrate the inner workings of the hierarchical network. The original input sinogram gradually transforms into the final reconstructed image through a number of intermediate representations. The intermediate images have non-isotropic voxel sizes with coarser resolution along the depth dimension (*t*) compared to the radial channel dimension (*r*). As reconstruction progresses, the intermediate images become more isotropic and the number projection angles (or the number of partial images) decreases, until the final reconstructed image is formed.

### Network structure

**Fig. 5:**
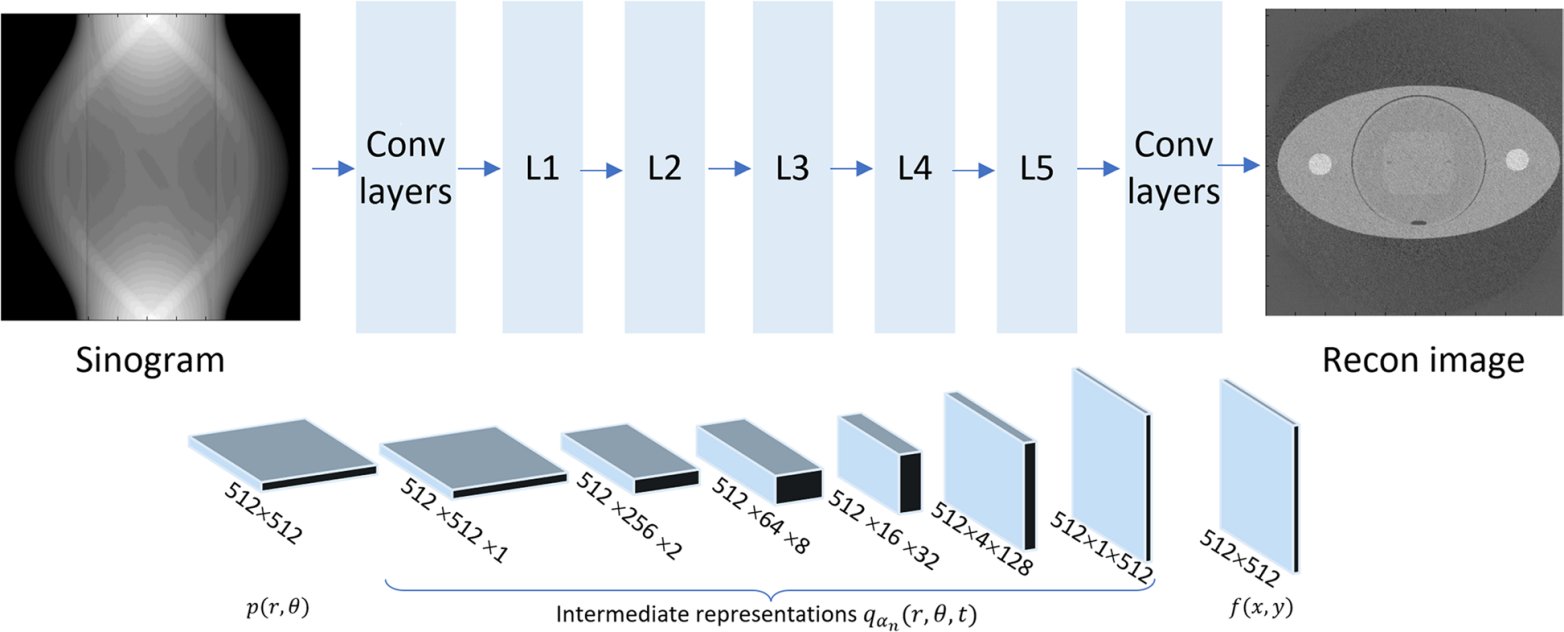
Network architecture for hierarchical CT reconstruction. Layers L1 through L5 are sparse connection layers that gradually transform the data from the sinogram domain to the image domain. Optional sinogram-, image-, or intermediate-domain convolutional layers can be incorporated to further optimize image quality

The results in Fig. 4 were generated by a feed forward network consisting of 5 hierarchical stages ($L1$ to $L5$). The overall network structure is illustrated in Fig. 5. The input sinogram contains 512 parallel-beam CT detector channels and 512 projection angles equally spaced over 360 degrees. The output image size is 512 × 512. As the reconstruction progresses through the hierarchy ($\mathrm{L}1$ through $\mathrm{L}5$), the number of angular bins is reduced, and the number of depth bins is increased, while the total size of data (#depths × #angles × #radial bins) remains constant. The data dimension at each hierarchical stage is shown in Table 1. These dimensions are chosen as an example illustration. For other CT geometries or when the sinogram dimension is not equal to the reconstructed image dimension, the specific data sizes in the radial, angular, and depth dimensions at each hierarchical stage should be properly chosen to allow a gradual transition from the sinogram dimension to the reconstructed image dimension. The ratio of the number of depth bins and projection angles between hierarchical stages do not have to be a power of two or an integer, because the network can be trained to perform resampling and interpolation of the data between hierarchical stages. The total size of data is preferably kept approximately constant across hierarchical stages to preserve all information from the original sinogram, but this is not a strict constraint. The implementation and evaluation of the hierarchical framework for more general data dimensions and fan- or cone-beam geometry can become an important topic of future study.

**Table 1:** Data dimension at each hierarchical stage

Hierarchical stage (L)	Data dimension		
	#depth bins (${N}_{t}$)	#proj. angles (${N}_{\theta }$)	#radial bins (${N}_{r}$)
L = 0 (line integrals)	1	512	512
L = 1	2	256	512
L = 2	8	64	512
L = 3	32	16	512
L = 4	128	4	512
L = 5 (image voxels)	512	1	512

In this initial study, the elementary partial reconstruction in each hierarchical stage ($\mathrm{L}1$ to $\mathrm{L}5$) is modeled as a linear transform. If each partial reconstruction were implemented by a fully connected network layer, this would require ${512}^{4}$ or 69 billion parameters per stage, which is unrealistic to implement and train. However, as mentioned earlier, the high dimensionality is overcome by the fact that the elementary partial reconstruction in each hierarchical stage is a local transform, thus most of the network weights are zeros and do not need to be stored or trained. In our implementation, $\mathrm{L}1$ through $\mathrm{L}5$ are implemented as customized “sparse connection” network layers, which are similar to fully connected layers but only non-zero network weights are stored and trained. More specifically, in the sparse connection layers, each input neuron is only connected to a small neighborhood of output neurons. In this work, we empirically chose the neighborhood size to be 3 depths $\times$ 5 angles $\times$ 3 radial bins. In the limiting case where the neighborhood of each neuron is expanded to the full output data (e.g., for L3, this means 32 depths $\times$ 15 angles $\times$ 512 radial bins), the sparse connection layer would become a fully connected layer. We used the sparse matrix structure provided by Tensorflow to represent these sparse connections. The network forward operator for the sparse connection layer is the product of the layer’s input data and the sparse matrix. Similarly, the network back propagation operator is the product of the transpose of the sparse matrix and the backpropagated gradient. We did not enforce rotational symmetry or other constraints in the non-zero network weights, making the sparse layers very flexible in expressing more general operations. It is worth noting that the sparse connection layer is different from a drop out layer, which removes output neurons instead of the connections between the input and output neurons of this layer.

Let *n* denotes the data dimension of the sparse connection layer, in this case $n=512\times 512$. The number of network parameters in each sparse connection layer is on the order of $O(n)$. Because the number of angles decreases exponentially from one hierarchical stage to the next, the total number of hierarchical stages is expected to be on the order of $O(\mathrm{log}n)$. Multiplying these two factors, a hierarchical reconstruction network overall would require a number of network parameters on the order of $O(n\mathrm{log}n)$. This contrasts with a generic fully connected network, which would require $O({n}^{2})$ parameters and become intractable for realistic data sizes. In our implementation, all sparse connection layers ($\mathrm{L}1$ to $\mathrm{L}5$) together used about 42 million trainable parameters, corresponding to only 0.06% of those in a fully connected layer of the same input and output dimensions (${512}^{4}$ or 69 billion parameters). For the 3D case, the number of parameters of a fully connected network would be on the order of $O({n}^{3})$ (${512}^{6}$ or 18 quadrillion), where we estimate – by extrapolation – the proposed approach would require on the order of $O({n}^{2}\mathrm{log}n)$ or 21 billion parameters.

In this initial study, the network layer at stages $\mathrm{L1}$ through $\mathrm{L}5$ used linear activation. The first layer was a sinogram domain convolutional layer with a filter kernel size of 512. The last four layers were image domain convolutional layers with 3 $\times$ 3 kernels and ReLU activation. Optional convolutional layers and non-linear activation could also be inserted between hierarchical stages, which could be a topic for a follow up study.

### Training

Mean squared error was used as the training loss. Stochastic gradient descent with a batch size of 20 was used. The sparse connections were initialized with all ones and convolution kernels were initialized with the Glorot uniform initializer. All networks were implemented in TensorFlow with Keras frontend. Training was performed with a NVIDIA Tesla V100 GPU.

**Fig. 6:**
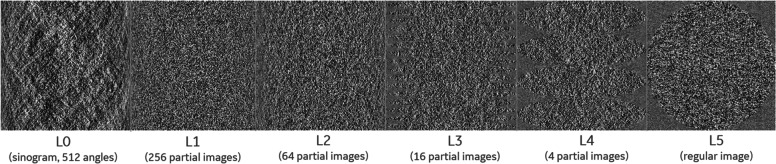
An instance of the noise pattern realizations used for training labels for intermediate representation levels. For better visualization, partial reconstructions of the same hierarchical stage (for different angles) are stacked together vertically

Training of the hierarchical network was first performed with 200 realizations of computer generated white Gaussian random noise patterns and their corresponding distance-driven [37] forward projections. These data pairs encode the tomographic transform and thus can be used for training the inverse transform. We also analytically generated intermediate training labels for each hierarchical stage (partial line integrals) by FBP reconstructions onto the corresponding non-isotropic voxel grids at this stage. An instance of the training labels is shown in Fig. 6. (Alternatively, these intermediate datasets could be computed by reprojecting the noise images over partial line integrals). The sparse connection layers ($\mathrm{L}1$ through $\mathrm{L}5$) were first pre-trained individually, then an end-to-end training of the entire network was performed (i.e., without intermediate training labels). The purpose of the pre-training is to initialize the weights in the sparse connection layers to a reasonable order of magnitude and reduce the total training time. The pre-training did not affect the loss function for the final end-to-end training. When trained end-to-end, to address the issue of vanishing gradients, a single network layer was updated at each training iteration, while the parameters of other network layers were frozen. The network layer being updated is randomly selected at each iteration. We did not include non-linear activation units in the network during the noise-based training, to constrain the network into learning a linear inverse operator without adversely learning the appearance of noise as prior information.

Following the noise-based training, the network was refined by an end-to-end training/validation with 200 clinical CT images. The training pairs were obtained by reprojecting the clinical CT images with the same parallel-beam geometry and inserting random noise to emulate additional measurement noise. The non-linear activation units were included in the convolutional layers during the training with clinical images to learn the non-linear prior information from the high-quality, low-noise clinical images. For testing, 50 additional clinical images were used, obtained the same way as the above training pairs, i.e., by reprojection and noise insertion. In this initial implementation, it took about 4 h to train the individual layers in the pre-training step, and it took about a day for the end-to-end refinement.

## Results

**Fig. 7:**
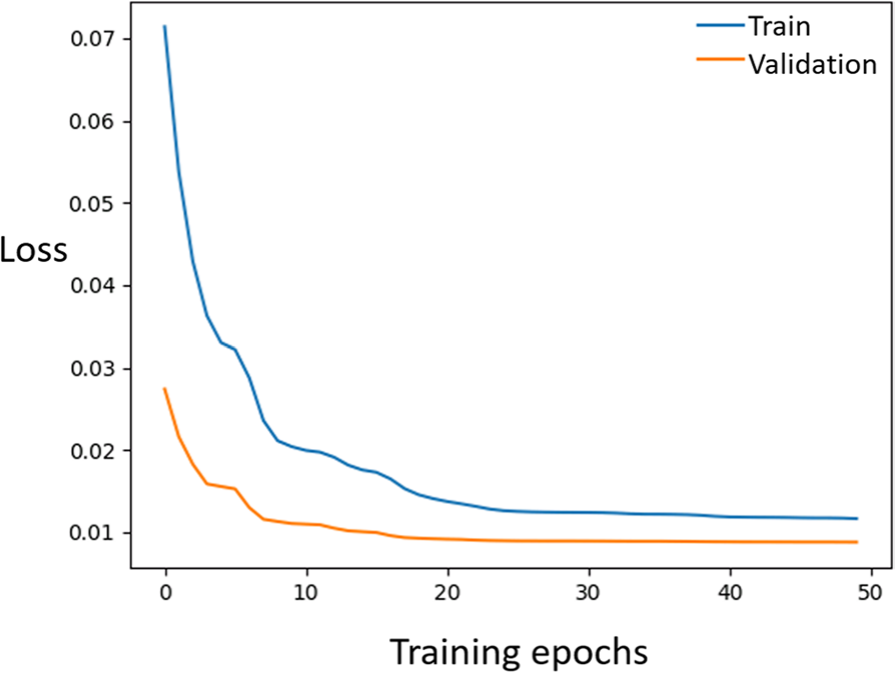
Loss function of the hierarchical network when trained end-to-end with pure noise. The validation loss is lower than the training loss because the validation dataset consists of clinical images, which contain more typical image features rather than random noise patterns

Figure 7 shows the loss function during end-to-end training with random noise patterns. Reasonable convergence behavior is observed for both the training and the validation data sets. The validation loss is lower than the training loss because the validation dataset consists of clinical images, which contain more regular image features instead of random noise patterns.

**Fig. 8:**
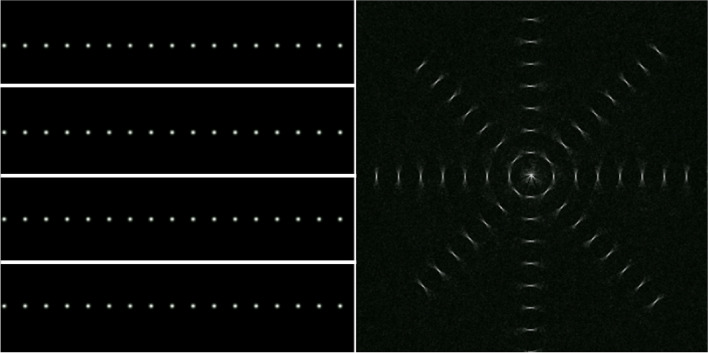
Visualization of the elementary reconstruction step at layer L5. Four partial images, each containing a row of points (left) were fed as input to layer L5 and the corresponding output (right) are displayed. Each point in the input is mapped to a small line segment corresponding to a partial line integral in the intermediate data domain

To visualize how the intermediate reconstruction steps work, an array of points was fed as the input to the sparse connection layer L5 (Fig. 8, left). These points are mapped to short line segments in various orientations at the output (Fig. 8, right). This is because each point at the input of L5 represents a partial line integral in the intermediate data domain. It also illustrates that the elementary reconstruction step has local-to-local correspondence between its input and output.

**Fig. 9:**
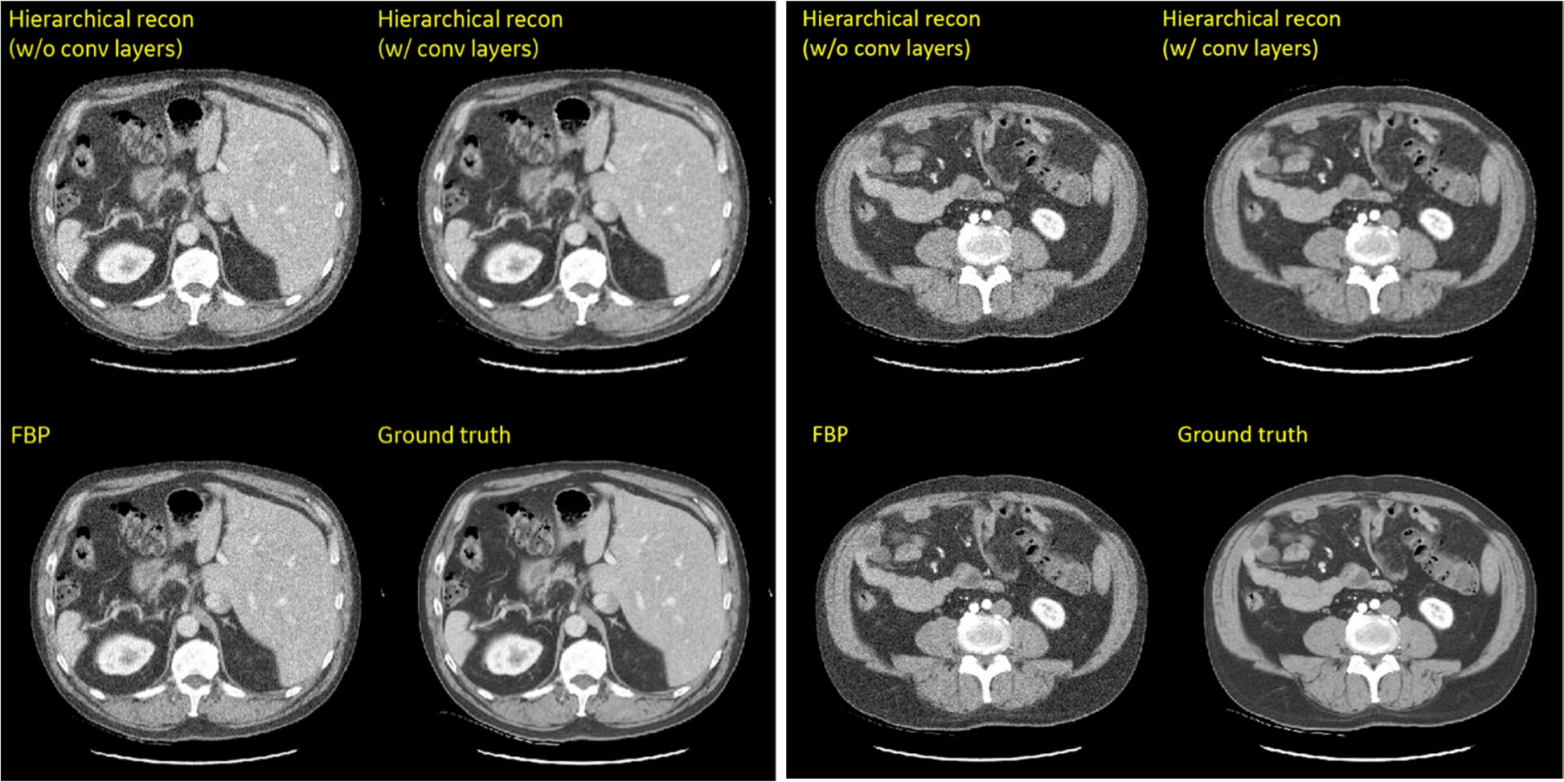
Examples images of hierarchical reconstruction in comparison with reference reconstructions. Two anatomical slices are shown. Window width/level = 400/0 HU

Figure 9 shows the final hierarchical DL reconstructions along with the corresponding FBP reconstructions and the original clinical images (prior to reprojection and noise insertion). Two versions of the hierarchical reconstruction, without and with the optional image-domain convolutional layers, were compared. The hierarchical DL without the convolutional layers were trained only with the simulated noise patterns (Fig. 6), without any prior information from the clinical images. This network is intended to show the feasibility of learning the CT reconstruction transform without any pre-conceived notion of emphasizing certain locations, frequencies or patterns. When tested with clinical CT images, the noise-trained hierarchical reconstruction without the convolutional layers is visually comparable to the FBP reference reconstruction, suggesting that the hierarchical network can effectively model the inverse Radon transform. In the second version of hierarchical reconstruction, convolutional layers were added to hierarchical DL network and further included clinical CT images in the training. As show in Fig. 9, the convolutional layers and additional training with clinical images further reduced noise in the hierarchical reconstruction relative to the FBP reconstruction.

## Discussion

The results illustrate the feasibility of hierarchical DL for CT reconstruction and also provide some intuition about its inner working. The hierarchical decomposition framework leads to a progressive transformation from many view angles to fewer view angles, while gaining depth resolution (Fig. 4). This illustrates the intuitive origin of the proposed approach, as humans also perceive some depth information from the limited stereoscopic range of the eyes [38]. Similarly, tomosynthesis imaging produces images with limited depth resolution from a limited angular view range [39]. The final hierarchical stage in our proposed framework is more commensurate with time-of-flight (TOF) PET reconstruction, where measurements with some level of depth resolution (depending on the TOF timing resolution) provide higher quality reconstruction than traditional PET measurements [40].

This hierarchical framework effectively localizes each phase of the reconstruction in the sense that the reconstructed variables in each hierarchical level only depend on a limited range of variables in the adjacent hierarchical levels, as illustrated in Fig. 8. Mathematically, this means that the respective matrix representations of the intermediate reconstruction steps are multiple orders of magnitude smaller than the inverse of the system matrix of the full forward model, which we believe is the key to making learnt reconstruction possible for realistic data sizes. While our work so far was limited to (full-size) 2D datasets, the framework is extendable to three dimensions. Because network connections between hierarchical levels are sparse, the computational complexity of our approach scales only linearly with data size, even for extension to three dimensions.

The image quality of the full-scale learnt CT reconstruction visually matches that of FBP reconstruction, a technique that has been the gold-standard for decades. The new approach has a long runway ahead in terms of possible improvements. In future work, image quality will be improved by building on the proposed hierarchical framework and incorporating more advanced network layers and training schemes, and further topics for development and evaluation may include more generic imaging geometries, metal artifacts in CT, and the data statistics and correction factors for emission reconstruction. We hope that it could eventually outperform state-of-the-art direct and iterative reconstruction techniques and combine the best of both other classes of reconstruction, i.e., exceed the image quality of iterative reconstruction at computation times below those of direct reconstruction.

We introduced the idea of training DL networks with computer simulated random noise patterns, overcoming the needs for large amount of clinical training data for supervised learning. The rationale for using random noise images is rooted in the robustness of training the network without any pre-conceived notion of emphasizing certain locations, frequencies or patterns. The counter-argument is that this noise-based training does not teach the networks any prior information about what clinical images look like. In fact, it may steer the network towards reconstructing noisy patterns, which is not a desired outcome. In other words, the noise-training approach is well-suited for training a network to robustly perform the inverse Radon transform. The incorporation of prior information has proven to be highly powerful, initially through Markov Random Field regularization techniques [41], later in approaches such as dictionary learning [42] and non-local means [43], and most recently in DL based priors [44–46]. Hence, we expect that the performance of the proposed hierarchical reconstruction will be enhanced by relying entirely on a wide variety of high-quality clinical images and corresponding noisy sinograms. Future research will include more extensive training, validation and comparison with state-of-the-art iterative reconstruction and more traditional DL techniques.

In this study, non-linear activation was only used in the convolutional layers at the end of the network. However, as a future topic, convolutional layers and non-linear activations can be inserted between the sparse connection layers to incorporate non-linear prior information into the intermediate reconstruction domains. We except this to further improve the image quality of the proposed hierarchical reconstruction.

A key ingredient for the implementation of the proposed hierarchical network is the sparse connection layers. These layers are a general tool for realizing transforms with a high degree of locality without using a fully connected layer. In this study the sparse connections were based on an empirically pre-determined neighborhood size in the intermediate data domain. The tradeoff between the neighborhood size (i.e., the sparsity of partial reconstruction steps) and the accuracy of reconstructed image may be evaluated in the future. Another future research topic is that, instead of using predetermined sparse connections, dynamic routing algorithms could be potentially used to prune or create connections during training and further optimize these connections.

While the hierarchical framework suggests rotational symmetries (Fig. 3), these were currently not yet explicitly exploited. In principle, DL network could be at one angle and redeployed at all angles. This should greatly improve training efficiency and further decrease network dimensionality. This may be achieved manually or through more implicit structural changes and will be an interesting area for future research.

**Fig. 10:**
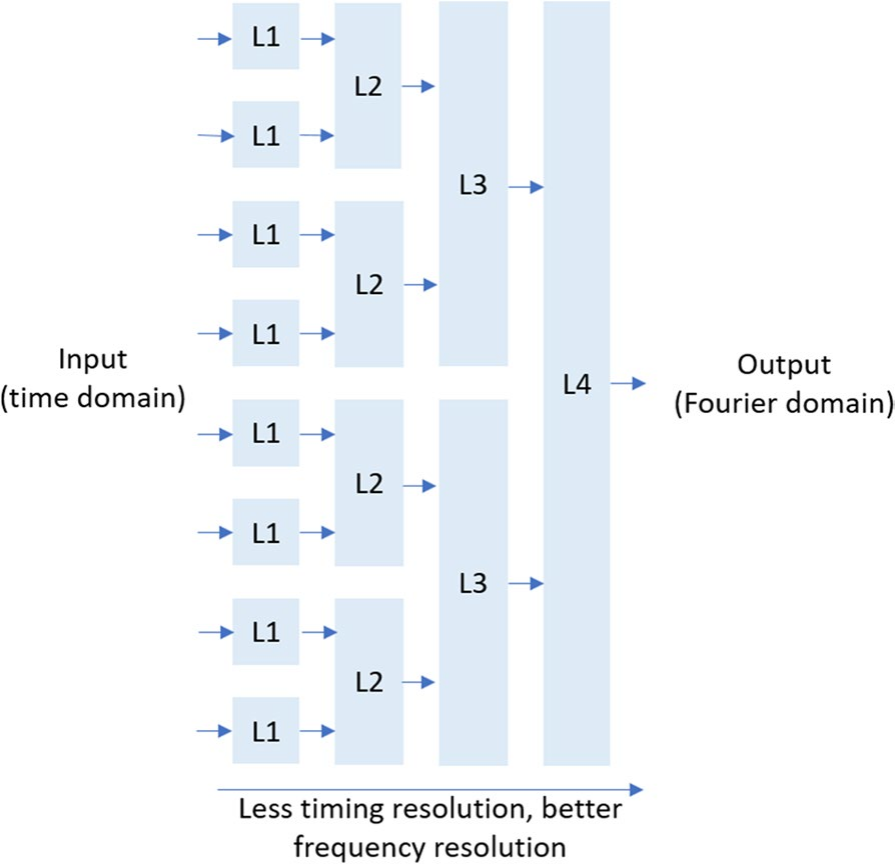
A hierarchical network to realize a Fourier transform. Each block represents a sparse network layer. The overall the dataflow resembles the classic radix-2 FFT

The proposed hierarchical framework is not limited to applications in CT reconstruction. Fourier-related transforms are another example that can be efficiently mapped to a neural network with the proposed hierarchical decomposition. To show this, one can express Fourier transform in the form of the Fredholm equation, with the integration kernel being $K\left(u,v\right)={e}^{-iuv}$. As shown in the previous analysis, a window function can be applied to the integral and define intermediate domains between input and output. Applying the window function to the Fourier transform gives rise to the short-time Fourier transform, thus the intermediate domain is essentially a time–frequency joint distribution of the input data. Due to the uncertainty principle, a wider time-window leads to coarser timing resolution but finer frequency resolution, and vice versa. By varying the window size one can obtain a progressive transform between the time- and frequency-domains, factorizing the Fourier transform into incremental steps, which can be more efficiently mapped to a deep neural network than a brute-force fully connected network model (Fig. 10). We further notice that if the window function is widened by a factor of two at each hierarchical stage, the resulting data flow will resemble the classic radix-2 fast Fourier transform (FFT) algorithm, thus should offer a similar order of savings in terms computational complexity. It is conceivable that the hierarchical framework can be adapted to Fourier-related reconstructions such as MRI reconstruction. Overall, it is possible to extend the hierarchical framework to a wide range of image reconstruction problems.

More broadly, the proposed methodology can be generalized to other transforms or inverse problems, where the hierarchy can be defined explicitly (such as through Fredholm’s equation) or implicitly (through end-to-end learning and using dynamic routing algorithms to prune out connections). Similarly, the approach can be used to solve large sets of equations and for numerical decomposition of large matrices.

## Conclusions

We have presented a hierarchical approach to DL which enabled purely data-driven supervised-learning of CT reconstruction from full-size 2D data without relying on conventional analytical or iterative reconstruction algorithm structures. Sparse connection layers were introduced to implement the hierarchical network and reduce the dimensionality of the tomographic inversion problem. The network was partially trained with random noise patterns that encode the transform of interest. The image quality of these first learnt reconstruction results matches that of FBP reconstruction. In terms of computational cost, the hierarchical approach required only $O(n\mathrm{log}n)$ parameters compared to $O({n}^{2})$ parameters as needed by a generic network, making the proposed approach scalable to large data dimensions. In theory such a hierarchical approach should require a smaller order of arithmetic operations than analytical FBP reconstruction. The method opens the door to an entirely new type of reconstruction, which – with further improvements – could potentially lead to a new breakthrough in the tradeoff between image quality and computational complexity. The proposed hierarchical decomposition framework can be extended to Fourier transforms and other tomographic reconstruction problems. More broadly, it is conceivable to generalize the same methodology for solving any large-scale inverse problems and matrix decompositions.

## Data Availability

All data generated or analyzed during this study are included in this published article.

## Abbreviations

DL: Deep learning
CT: Computed tomography
FBP: Filtered backprojection
2D: Two-dimensional
TOF: Time-of-flight
FFT: Fast Fourier transform
